# Effect of Music on Patient Experience during Intravitreal Injection

**DOI:** 10.1155/2020/9120235

**Published:** 2020-07-30

**Authors:** Jonathan Cheuk-hung Chan, Lok Pong Chan, Chi Pong Yeung, Tin Wai Tang, Yip Mang O, Wai Ching Lam

**Affiliations:** ^1^Department of Ophthalmology, LKS Faculty of Medicine, University of Hong Kong, Pok Fu Lam, Hong Kong; ^2^LKS Faculty of Medicine, University of Hong Kong, Pok Fu Lam, Hong Kong

## Abstract

**Introduction:**

Many patients remain anxious during intravitreal injections, despite its increasing use. As music can alleviate anxiety for other procedures, we wanted to evaluate its effect during intravitreal injection.

**Methods:**

Patients undergoing routine intravitreal injection were recruited for a randomized controlled trial. Subjects complete a State-Trait Anxiety Inventory (STAI-S) questionnaire before and after undergoing injection with or without background music. They were also assessed for subjective satisfaction, anxiety, pain, and future preferences after the injection.

**Results:**

There were 39 and 37 Chinese subjects in the music (age 68.08 ± 13.67) and control (age 73.24 ± 11.17) groups, respectively. The music group reported lower anxiety and pain, and a greater reduction in STAI-S score, but the differences were not statistically significant (*P* = 0.830, 0.655, 0.199, respectively). More subjects in the music group (92.3%, control group 64.9%) preferred music for future injections (*P* = 0.003). Age, but not the number of previous injections, was negatively correlated with reported anxiety (*r* = −0.27, *P* = 0.021).

**Conclusion:**

Most subjects preferred music during future injections. Although music reduced anxiety, the effect was not statistically significant and may be masked by the higher age of our control group, as increasing age was correlated with lower anxiety.

## 1. Introduction

For many years prior to the introduction of antivascular endothelial growth factor (anti-VEGF) agents, intravitreal injections were performed sporadically, mainly for the delivery of antibiotics in bacterial endophthalmitis. Since the popularization of anti-VEGF therapy in the past decade, increasing number of intravitreal injections are now being performed regularly for the treatment of various retinal disorders. These include age-related macular degeneration, retinal vein occlusion, and diabetic macular edema. Often performed as an outpatient procedure, intravitreal injection is fast, convenient, and relatively safe. Due to its increasing utilization in many centres around the world, there has been an increased focus on the patient experience for this procedure, considering that some apprehension and discomfort associated with needle penetration of the globe is probably expected. One early randomized controlled study with 93 subjects had examined the role of a different topical anesthetic regime on perceived pain and noted that the pain was mild irrespective of the anesthetic method used [1]. However, another study which examined both anxiety and pain in 225 subjects receiving intravitreal injections [2] reported that 25% had a Visual Analogue Scale for anxiety level of 6 or greater (out of a maximum of 10), which, in turn, was correlated with increased pain perception. It is likely that reducing anxiety may improve patient experience aside from its possible benefit on the perceived pain, as reported in a study of 300 subjects with wet age-related macular degeneration, where anxiety about the intravitreal injections was much more common than the actual perceived pain from the procedure [3]. With the increasing number of injections being performed and the recognition of significant anxiety during the procedure, there have been various studies investigating different methods to alleviate anxiety and improve patient experience. Playing music during the procedure is one possible method. Use of music to reduce anxiety and pain has been previously reported for cataract surgery. A study of 121 randomly selected elderly patients [4] found that relaxing music during surgery led to a more satisfying experience. While several studies have shown similar results for cataract surgery [5–7], the benefits of music for intravitreal injection have not been well studied. Currently, there is only one randomized clinical trial conducted by Chen which suggested music could play a significant role in reducing anxiety during intravitreal injections, as reflected from the decrease in the anxiety level as measured by the state anxiety portion of the Spielberger State-Trait Anxiety Inventory (STAI-S) [8].

Various types of music have been used in previous studies of their effect on patient experience in cataract surgery, including “classical music accompanied by soothing sounds of nature”, meditation music, and Korean traditional instrumental music [4, 6, 7]. The Korean study highlighted a possible cultural factor on the type of music in different populations [7]. In this regard, it should be noted that Chen's study on intravitreal injections was conducted in the USA with background Western classical (Mozart) music [8] and may not reflect the experience elsewhere, due to possible cultural differences in music preferences.

To the best of our knowledge, no previous studies have been conducted on the effect of music on patients' experience during intravitreal injections in Hong Kong. Therefore, this study aims to evaluate the possible benefit of music in terms of the patient's satisfaction, pain, and anxiety levels during intravitreal injections.

## 2. Materials and Methods

This two-armed, parallel-design randomized controlled trial was approved by the Institutional Review Board of the University of Hong Kong/Hospital Authority Hong Kong West Cluster (HKU/HA HKW IRB; reference number UW 18–564), and adhered to the tenets of the Declaration of Helsinki. In addition, the study was registered in the publicly assessable University of Hong Kong Clinical Trials Registry (HKUCTR), at http://www.HKUCTR.com (Study Identifier: HKUCTR-2595). Scheduled intravitreal injections at our centre (Grantham Hospital, Hong Kong) are generally conducted within an operating room at the Day Surgery Centre. During the study recruitment period of January to March 2019, patients attending for scheduled, elective, intravitreal anti-VEGF injection were identified and given information on the study. Those who agreed to participate were recruited as subjects after they had given their informed consent in writing. Patients who were incapable of giving informed consent, illiterate or cognitively impaired, deaf or using hearing aids, or with known psychological and psychiatric disorder were excluded from the study.

For the sample size calculation, we referenced Chen's previous study [8] where a 10% difference was noted in the mean reduction of the STAI-S score after intravitreal injections (compared to before injections) for the music therapy group (decrease of 19%, *n* = 37) and control group (decrease of 9%, *n* = 36). To allow for the possibility of a smaller difference in our subjects, we set a difference of 5% between the 2 groups as being clinically significant, giving a target sample size of 37 for each group when the statistical significance is *α* = 0.05 and the power is 1 − *β* = 0.8.

**Figure 1:**
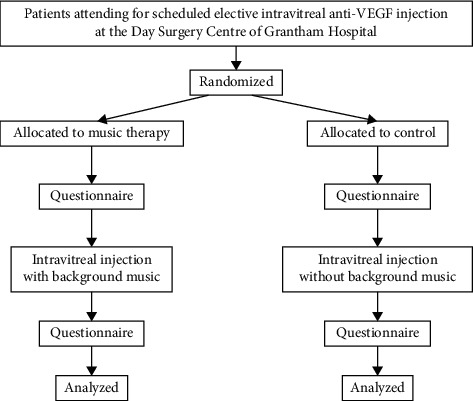
Flow diagram of the recruitment, randomization, evaluation, and analysis process.

All subjects undergoing intravitreal injection during the study period will be randomly assigned to intravitreal injection with either music (“music therapy”) or no music (“control”) in the operating room (shown in Figure 1). Subjects are not informed of their assigned intervention prior to completing an STAI-S questionnaire and entering the operating room. Subjects randomized to the music therapy group underwent the procedure with background music playing in the operating room, while subjects assigned to the control group underwent the same procedure without any background music playing. The background music chosen was *Relaxing Piano: Studio Ghibli Complete Collection* (Cat Trumpet, 2017. *Spotify,* spotify: album: 3lhu4QblOSyvjU8tR4RgwM). The type of music chosen was guided by a small prestudy survey of 12 patients from different age groups (3 aged 50–59; 2 aged 60–69; 3 aged 70–79; and 4 aged 80–89) during 2 injection sessions conducted in December 2018. These 12 patients were generally receptive to background music during their procedure, with soothing and easy-listening instrumental being the most preferred type. We, then, ran a trial run for these 12 patients using music eventually chosen for this study, and all responded positively to the experience. For the study, music is played continuously throughout the procedure for subjects in the music therapy group, while undergoing standard intravitreal injections as per our usual practice for nonstudy patients, including the application of topical anaesthesia and use of lid speculum. After the intravitreal injection, subjects completed another set of an STAI-S questionnaire and recorded their perceived pain, anxiety, and satisfaction for the procedure on a visual analog scale (VAS).

### 2.1. STAI-S Anxiety Score

**Figure 2:**
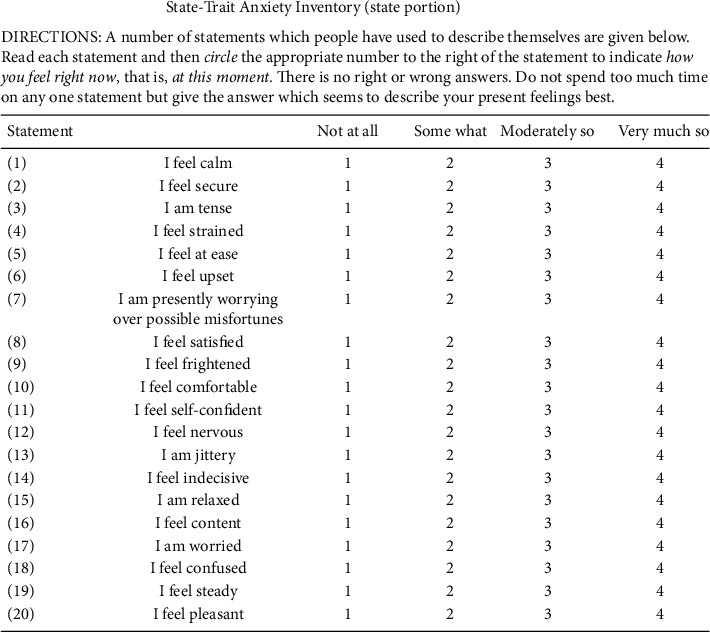
State portion of the Spielberger State-Trait Anxiety Inventory (STAI-S).

The state anxiety portion of the Spielberger State-Trait Anxiety Inventory (STAI-S) was used to evaluate the anxiety level of the subjects before and after the injections (shown in Figure 2). This test has 20 statements about the subject's current emotional state (for example, “I am relaxed” and “I feel nervous”), with 4 possible answers (select one) which best describe their feeling for each statement (“Not at all,” “Somewhat,” “Moderately so,” and “Very much so”). The answers would be converted to a score from 20 to 80, with 20 indicating the lowest anxiety level and 80 as the highest level. The average score before and after the procedure will be compared and analyzed between the music and control group.

### 2.2. VAS Questionnaire

**Figure 3:**
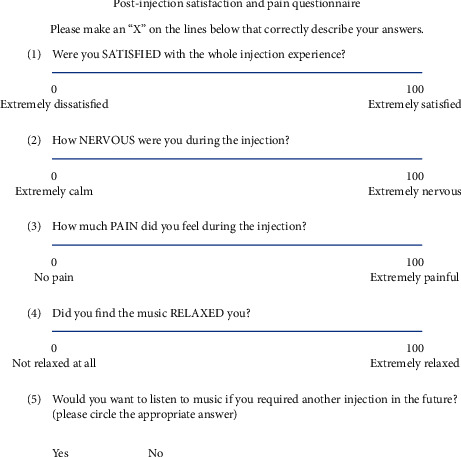
VAS questionnaire.

A questionnaire based on a visual analog scale (VAS) was created to evaluate the subjects' experience during the intravitreal injection (shown in Figure 3). The questions include

1. Were you SATISFIED with the whole injection experience?
2. How NERVOUS were you during the injection?
3. How much PAIN did you feel during the injection?
4. Did you feel the music RELAXED you? (if randomized to music therapy)

(Subjects will give a score from 0 to 100 for each of the abovementioned questions).

A yes/no question to evaluate their preference for subsequent intravitreal injections:

(5) Would you want to listen to music if you required another injection in the future?

The age, number of previous injections, blood pressures, heart rate, STAI-S, and VAS scores were analyzed with an independent *T*-test, while the gender and preference for music during subsequent injections were analyzed with the chi-square test. Pearson's correlation coefficient was used to measure the linear correlation between age and number of previous injections, with change in the STAI-S score (before and after the injection), reported pain and anxiety.

## 3. Results

A total of 78 subjects were initially recruited from January to March 2019, but 2 subjects in the music group were subsequently excluded as they had failed to hear any music in the operating room (OR). This leaves a total of 76 subjects for data analysis.

There were 39 (23 males, 16 females) subjects in the music therapy group and 37 (18 males, 19 females) subjects in the control group. All subjects were Chinese. The mean age, number of previous intravitreal injections, and baseline STAI-S score for the music and control group were 68.08 ± 13.67 and 73.24 ± 11.17, 9.44 ± 7.70 and 8.35 ± 9.25, and 39.41 ± 12.12 and 35.16 ± 10.34, respectively (shown in Table 1). No significance differences were noted between the 2 groups for age, sex, the number of previous injections, and the baseline STAI-S score. The baseline heart rate, systolic blood pressure (SBP), and diastolic blood pressures (DBP) in the music and control group were 77.51 ± 12.17 and 78.70 ± 13.45 beats per minute (BPM), 149.74 ± 27.98 and 152.00 ± 34.71 mmHg, and 79.26 ± 10.22 and 80.76 ± 18.75 mmHg, respectively, with no significant differences between the 2 groups (shown in Table 1).

After injection, both the music and control group reported (on the VAS) a similar level of satisfaction (86.28 ± 13.99 and 87.73 ± 18.62, respectively), but slightly less anxiety (36.79 ± 32.01 versus 38.51 ± 37.62, respectively) and pain (31.92 ± 28.30 versus 34.95 ± 30.49, respectively) was reported by the music group, although the differences were not statistically significant (shown in Table 2). Subjects in the music group generally reported being relaxed by the background music during their injection procedure in the OR (70.33 ± 23.84). Regarding future intravitreal injections, overall, most subjects preferred having background music, although this was significantly higher in the music group compared to the control group, at 92.3% and 64.9%, respectively (*P*=0.003). In addition, there was a greater mean reduction in the STAI-S anxiety score in the music group (decreased 9.13 from baseline) compared to the control group (decreased 6.46 from baseline), but this was not statistically significant (*P*=0.287). In terms of SBP, DBP, and heart rate changes after the intravitreal injections, no significant difference was found between the 2 groups (shown in Table 2).

Pearson correlations did not show any significant relationship between the number of previous injections and postinjection anxiety (*r* = 0.06, *P*=0.633), pain (*r* = −0.14, *P*=0.235), or STAI-S changes (*r* = 0.12, *P*=0.288). There was also no correlation between age and postinjection pain (*r* = −0.10, *P*=0.369) or STAI-S changes (*r* = −0.12, *P*=0.303), although it was negatively correlated with anxiety (*r* = −0.27, *P*=0.021).

## 4. Discussion

To our knowledge, this is the first randomized control study to evaluate the effects of music on intravitreal injections in an Asian (Chinese) population. Similar to the study conducted at the Yale Eye Centre in Connecticut, USA [8], where Western classical music was used exclusively, the results showed a larger reduction of anxiety, in terms of the STAI-S score, in subjects exposed to music during their intravitreal injection compared to the control group, although this was not statistically significant in our study. Both self-reported anxiety and pain also appear lower in the music group compared to the control group, but again, this did not reach statistical significance. In addition, most subjects exposed to music during the procedure found it relaxing. Although all subjects had a similarly high satisfaction rate whether music was played or not in this study, most would still prefer to have music for their next intravitreal injection, especially if they had been assigned to the music therapy group in this study. This preference for music is again similar to the finding in another randomized study [8], albeit in a different population of likely varying racial and cultural background (the racial/cultural background of the subjects were not specified in that study conducted at New Haven, Connecticut, USA).

We had expected that a history of previous injections would lessen intraoperative anxiety or pain, but was unable to find any significant correlation in our subjects. This finding is similar to that from an earlier report from the UK, where significant anxiety associated with intravitreal injections persisted irrespective of the number of previous injections received by the patient [3]. However, we did note that increasing age was significantly correlated with the decreasing level of reported anxiety.

Postoperatively, most subjects in our study would have preferred listening to music in the OR during their intravitreal injection. The reason for this preference was not readily apparent in this study, although there was a trend showing lower anxiety and pain perception among those who listened to music during their procedure compared to those who did not. We may need a larger sample size to show any significant differences in the parameters we have chosen, or there could be other, yet to be determined, reasons for patients to prefer music during their injections.

The beneficial effect of music, as suggested by this local study on Chinese subjects and the one conducted in the USA [8], is not limited to intravitreal injections, but extends to cataract surgery [4, 6, 7, 9] and other nonocular clinical procedures including mechanical ventilation, positron emission tomography scan, and gynaecological operation [10–12]. Besides benefiting the patients, music generally also helps to relax and calm the OR staff and may even enhance their performance, including the surgeon's [13–15]. None of the staff or subjects in our study reported any negative impact or adverse effect (for example, on communication) from having music playing in the OR during the injection procedure.

The absence of significant differences among our subjects in most of the assessed parameters (including anxiety and pain) may be due to the short procedural duration of intravitreal injections (range 6.75 to 18.4 minutes), such that any impact will be limited unless music exposure is extended to the immediate preoperative period as is the case with the study by Chen et al. [8], which was not feasible in our setting due to a large common waiting area for all patients (not just those awaiting intravitreal injections) at our Day Surgery Centre. In addition, the age of our subjects ranged widely from 29 to 97, so their music preference may vary, although our preliminary survey had shown a wide acceptance for soft, instrumental music (the chosen music for this study) among local patients undergoing intravitreal injections. Related to this, as increasing age was significantly correlated with lower reported anxiety, some beneficial effect of music therapy may have been masked by the higher mean age of our control group compared to the music therapy group, although the intergroup age difference was not statistically significant. We suggest that future studies should include a greater number of younger subjects, as well as using individual headphones in the waiting area, to better evaluate the effect of music on patient experience. Overall, most of our subjects (all local Chinese) preferred having intraoperative music for future intravitreal injections, but this was significantly greater among those randomized to have soft, instrumental background music during their procedure. We also noted a trend among subjects who listened to music for lower reported anxiety and pain and a higher reduction of anxiety from baseline, despite none of these differences reaching statistical significance.

## 5. Conclusions

Our study showed that increasing age was significantly correlated with decreased anxiety but the number of previous injections was not. The vast majority of our subjects preferred having music during their intravitreal injections, which showed a trend, though not significant, for improving anxiety and experience of the procedure. This is consistent with previous reports on music therapy for other procedures, although the type of music used and preferred may vary among different populations and cultures.

## Figures and Tables

**Table 1:** Baseline parameters.

	Music group (*n* = 39)	Control group (*n* = 37)	*P* value
Age (years)	68.08 ± 13.67	73.24 ± 11.17	0.076

Gender (male : female)	23 : 16	18 : 19	0.367

Number of previous injections	9.44 ± 7.70	8.35 ± 9.25	0.580

*Baseline assessments:*			
(1) STAI-S score	39.41 ± 12.12	35.16 ± 10.34	0.105
(2) Systolic BP (mmHg)	149.74 ± 27.98	152.00 ± 34.71	0.755
(3) Diastolic BP (mmHg)	79.26 ± 10.22	80.76 ± 18.75	0.669
(4) Heart rate (BPM)	77.51 ± 12.17	78.70 ± 13.45	0.687

**Table 2:** Comparison of outcomes.

	Music (*n* = 39)	Control (*n* = 37)	*P* value
*VAS score:*			
(1) Satisfaction	86.28 ± 13.99	87.73 ± 18.62	0.702
(2) Anxiety (nervousness)	36.79 ± 32.01	38.51 ± 37.62	0.830
(3) Pain	31.92 ± 28.30	34.95 ± 30.49	0.655
(4) Relaxed by music	70.33 ± 23.84		

*Postinjection changes:*			
(1) STAI-S score	−9.13 ± 10.51	−6.46 ± 6.98	0.199
(2) Systolic BP (mmHg)	13.44 ± 31.90	10.41 ± 30.59	0.674
(3) Diastolic BP (mmHg)	0.90 ± 15.90	−0.24 ± 15.24	0.751
(4) Heart rate (BPM)	−1.38 ± 17.59	−5.70 ± 7.33	0.165

*Preferred music for future injection(s)*	36 (92.3%)	24 (64.9%)	∗0.003

## Data Availability

The data that support the findings of this study are available from the corresponding author, JCH, upon reasonable request.
